# Automated phenotyping of plant shoots using imaging methods for analysis of plant stress responses – a review

**DOI:** 10.1186/s13007-015-0072-8

**Published:** 2015-04-17

**Authors:** Jan F Humplík, Dušan Lazár, Alexandra Husičková, Lukáš Spíchal

**Affiliations:** Department of Chemical Biology and Genetics, Centre of the Region Haná for Biotechnological and Agricultural Research, Faculty of Science, Palacký University, Šlechtitelů 11, Olomouc, CZ-78371 Czech Republic; Department of Biophysics, Centre of the Region Haná for Biotechnological and Agricultural Research, Faculty of Science, Palacký University, Šlechtitelů 11, Olomouc, CZ-78371 Czech Republic

**Keywords:** Plant phenotyping, RGB digital imaging, Chlorophyll fluorescence imaging, Thermal imaging, Hyperspectral imaging, Shoot growth, Biomass production

## Abstract

Current methods of in-house plant phenotyping are providing a powerful new tool for plant biology studies. The self-constructed and commercial platforms established in the last few years, employ non-destructive methods and measurements on a large and high-throughput scale. The platforms offer to certain extent, automated measurements, using either simple single sensor analysis, or advanced integrative simultaneous analysis by multiple sensors. However, due to the complexity of the approaches used, it is not always clear what such forms of plant phenotyping can offer the potential end-user, i.e. plant biologist. This review focuses on imaging methods used in the phenotyping of plant shoots including a brief survey of the sensors used. To open up this topic to a broader audience, we provide here a simple introduction to the principles of automated non-destructive analysis, namely RGB, chlorophyll fluorescence, thermal and hyperspectral imaging. We further on present an overview on how and to which extent, the automated integrative in-house phenotyping platforms have been used recently to study the responses of plants to various changing environments.

## Introduction

**Figure 1:**
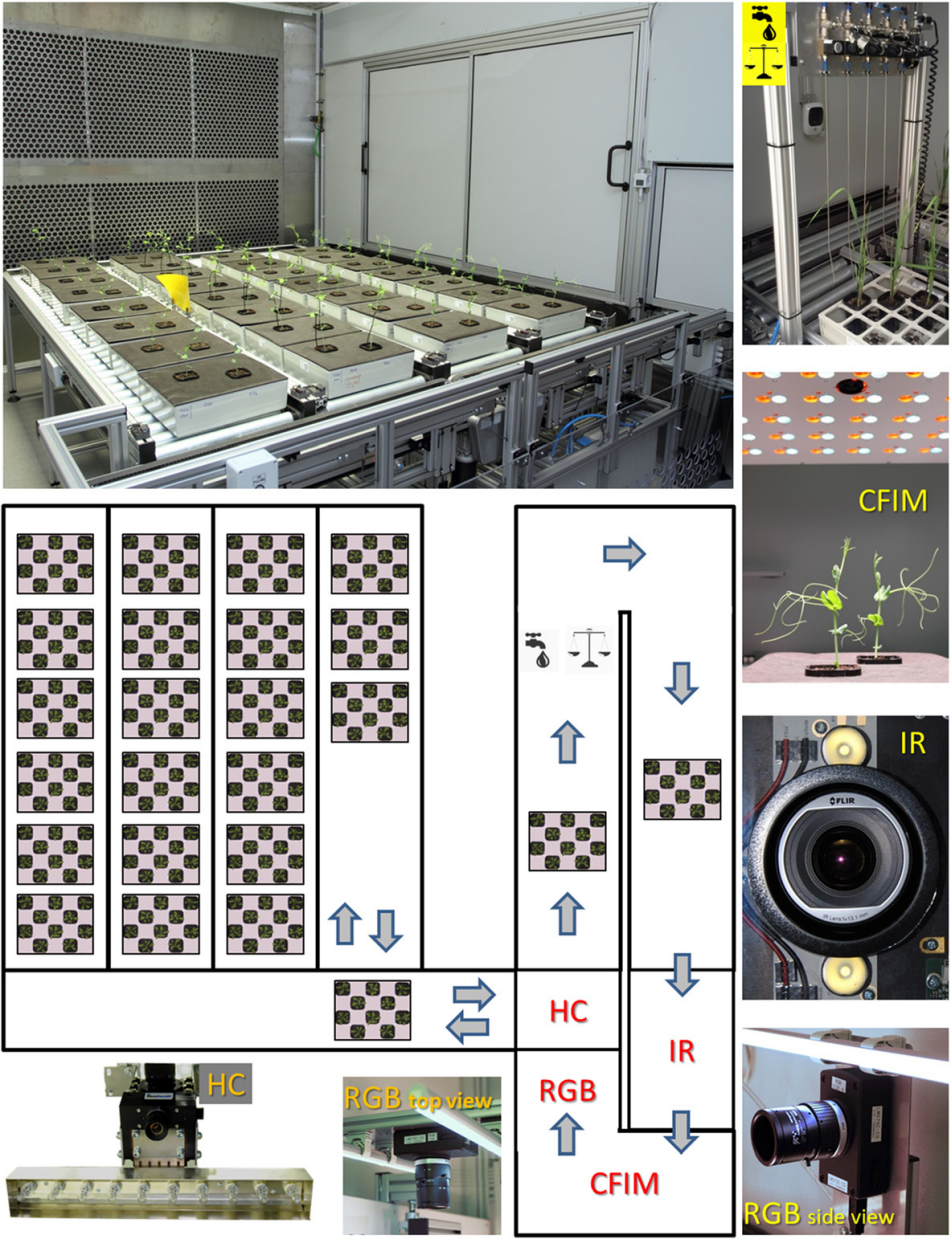
Scheme of the grow chamber-based automated high-throughput phenotyping platform PlantScreen™ (Photons Systems Instruments, Brno, Czech Republic), installed at Palacký University in Olomouc, Czech Republic [20]. The system is located in a growth chamber with white LED illumination (max. 1000 μmol photons m^−2^ s^−1^) and controlled environment (10 – 40°C, 30 – 99% relative humidity). The growth area with roller conveyer has capacity of up to 640 *Arabidopsis*, cereals and other crops grown in standardized pots. The measuring cabinet contains acclimation chamber for dark adaptation of plants coupled with an automated weighting and watering area. The cabinet is equipped with KCFIM and RGB imaging (top and 2 side views), thermoimaging (IR) to measure stomata openness and SWIR hyperspectral imaging to determine water content. The platform can be controlled either from the place or *via* remote control software. The operating software enables automatic data evaluation.

Recently, a large number of reviews have been published on the advantages and possibilities of high-throughput plant phenotyping approaches [1-5]. Most focus on the potential of these approaches which use precise and sophisticated tools and methodologies to study plant growth and development. To review the state-of-the-art of phenotyping platforms, we present a list of recent publications in Table 1. Interestingly, in about a half of these, only one measuring tool, mostly RGB imaging, for plant phenotyping was used. In the other papers, integrative phenotyping, signifying two or more measuring tools but which are rarely automated, was used (Table 1). This illustrates that the integrative automated high-throughput phenotyping measurements/platforms are still rather rare. Greenhouse- and grow chamber-based plant phenotyping platforms are publically available and these offer their services and collaborative projects. Descriptions, methodological background and focus can be found at http://www.plant-phenotyping-network.eu/eppn/select_installation. As an example of the integrative automated high-throughput phenotyping platform, a grow chamber-based phenotyping facility installed at Palacký University in Olomouc, Czech Republic is presented in Figure 1.

**Table 1:** **List of selected works describing automated high-throughput analysis to study plant stress responses**

**Study**	**Plant species**	**Type of stress**	**Type of the study**	**Type of automated analysis**	**Platform name/origin**
Granier et al. 2006; [58]	*Arabidopsis*	drought-stress	methodology	RGB (top view)	PHENOPSIS
Skirycz et al. 2011; [59]	*Arabidopsis*	drought-stress	applied	RGB (top view)	WIWAM
Clauw et al. 2015; [60]	*Arabidopsis*	drought-stress	applied	RGB (top view)	WIWAM
Tisné et al. 2013; [61]	*Arabidopsis*	drought-stress	applied	RGB (top view)	PHENOSCOPE
Neumann et al. 2015; [26]	barley	drought-stress	methodology	RGB (multiple views)	LemnaTec
Pereyra-Irujo et al. 2012; [62]	soybean	drought-stress	methodology	RGB (two-views)	GlyPh (self-construction)
Honsdorf et al. 2014; [16]	barley, (wild species)	drought-stress	applied	RGB (multiple views)	LemnaTec
Coupel-Ledru et al. 2014; [63]	grapevine	drought-stress	applied	RGB (multiple views)	LemnaTec
Petrozza et al. 2014; [66]	tomato	drought-stress	applied	RGB (multiple views), hyperspectral NIR, SLCFIM	LemnaTec
Harshavardhan et al. 2014; [67]	*Arabidopsis*	drought-stress	applied	RGB (top view), hyperspectral NIR	LemnaTec
Bresson et al. 2013; [68]	*Arabidopsis*	drought-stress	applied	RGB (top view)	PHENOPSIS
Bresson et al. 2014; [69]	*Arabidopsis*	drought-stress	applied	RGB (top view), TLCFIM	PHENOPSIS
Chen et al. 2014; [64]	barley	drought-stress	methodology	RGB (multiple-views), hyperspectral NIR, SLCFIM	LemnaTec
Fehér-Juhász et al. 2014; [19]	wheat	drought-stress	applied	RGB (multiple views), thermoimaging	self-construction, semi-automated
Cseri et al. 2013; [65]	barley	drought-stress	methodology	RGB (multiple views), thermoimaging	self-construction, semi-automated
Vasseur et al. 2014 [71]	*Arabidopsis*	heat-stress, drought-stress	applied	RGB (top view)	PHENOPSIS
Rajendran et al. 2009; [73]	wheat	salt-stress	applied	RGB (multiple views)	LemnaTec
Harris et al. 2010; [74]	wheat, barley	salt-stress	applied	RGB (multiple views)	LemnaTec
Golzarian et al. 2011; [18]	barley	salt-stress	methodology	RGB (multiple views)	LemnaTec
Schilling et al. 2014; [75]	barley	salt-stress	applied	RGB (multiple views)	LemnaTec
Hairmansis et al. 2014; [76]	rice	salt-stress	applied	RGB (multiple views) SLCFIM	LemnaTec
Chaerle et al. 2006; [77]	tobacco	biotic-stress	methodology	thermoimaging, TLCFIM	self-construction
Poiré et al. 2014; [79]	*Brachypodium*	nutrient-deficiency	methodology	RGB (multiple views )	LemnaTec
Neilson et al. 2015; [80]	*Sorghum*	nutrient-deficiency	methodology	RGB (multiple views ), hyperspectral NIR	LemnaTec
Chaerle et al. 2007; [81]	bean	nutrient-deficiency, biotic-stress	methodology	RGB (top view), thermoimaging, TLCFIM	self-construction
Jansen et al. 2009; [37]	*Arabidopsis*, tobacco	drought-stress, chilling-stress	methodology	RGB (top view), KCFIM	GROWSCREEN (self-construction)
Humplík et al. 2015; [20]	pea, field cultivars	cold-stress	methodology	RGB (multiple views), KCFIM	PlantScreen

High-throughput integrative phenotyping facilities provide an opportunity to combine various methods of automated, simultaneous, non-destructive analyses of plant growth, morphology and physiology, providing a complex picture of the plant growth and vigour in one run, and repeatedly during the plant’s life-span. Particular methods used in integrative plant phenotyping are often not new and usually represent those which have already been used for a number of years in basic research, e.g. non-invasive methods that employ visible or fluorescence imaging (described in more detail further in the text). High-throughput then allows analysis of the plants on a large scale. This enables users to apply statistics to discover subtle but significant differences between the studied genotypes and treatment variants.

The potential users of such facilities, mostly biologists, are often not very familiar with the applied physical methods used in integrative plant phenotyping. Thus, in this mini-review, we present a simple introduction to the basis of various non-invasive sensors used in high-throughput phenotyping platforms, namely visible red-green-blue (RGB) imaging, chlorophyll fluorescence imaging (CFIM), thermoimaging, and hyperspectral imaging. Further, we describe potential applications of some of the phenotyping methods that have been used to study the responses of different plant species to various stresses.

### Non-destructive analysis of growth and physiology of plant shoots

The methods for automated phenotyping and their aims have been reviewed in a number of recent reports [3,6,7]. In the following text we give a description of the basis of the automated non-invasive analysis of plant shoots and appropriate sensors that have been used for studies of plant stress responses.

#### Visible RGB imaging of plant shoots

**Figure 2:**
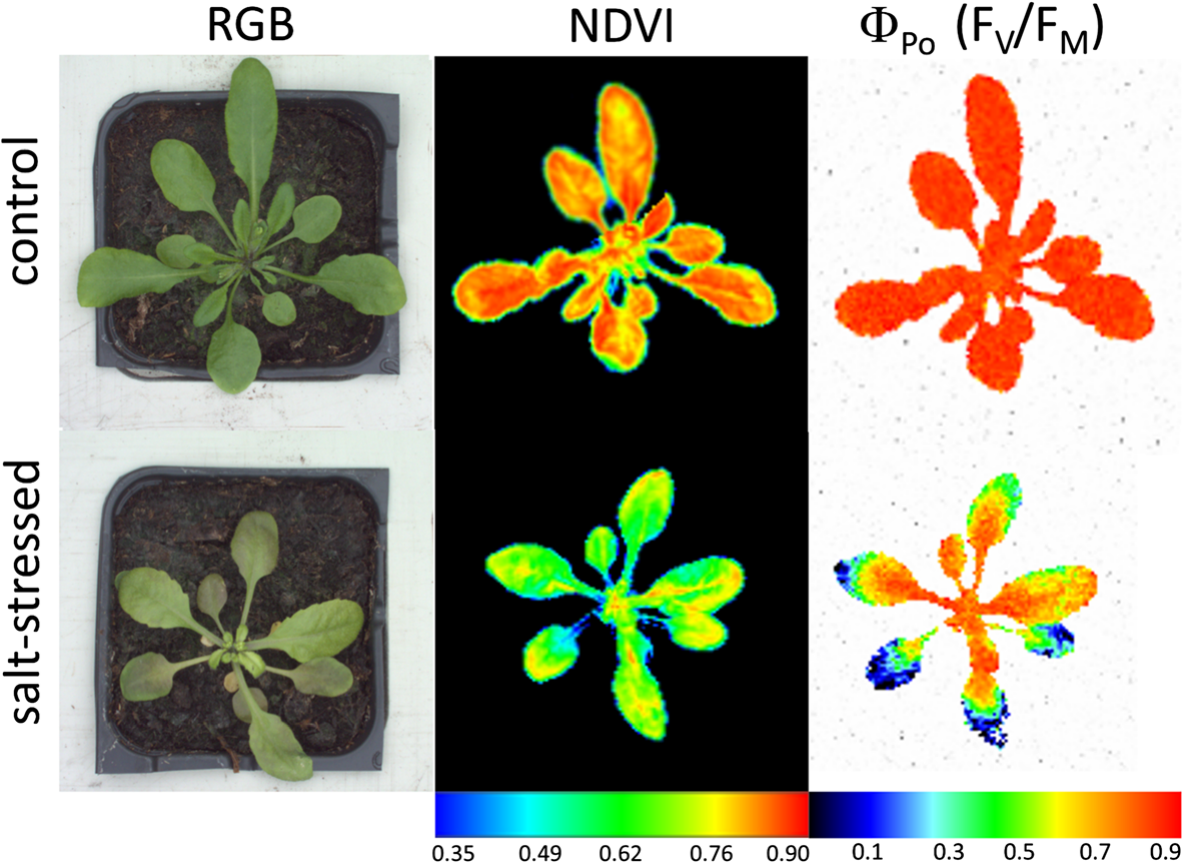
The illustrative figure presenting outcome of simultaneous analysis of control and salt-stressed *Arabidopsis* plants, using RGB, hyperspectral and Chl fluorescence imaging. The 18 DAG old soil-grown *Arabidospis* plants were treated with 250 mM NaCl (salt-stressed) and water (control) and after 48 hours were analysed by different sensors for comparison in: morphology (top-view RGB imaging can be used for computation of rosette area or shape parameters), spatial distribution of vegetation index reflecting changes in the chlorophyll content (NDVI) provided by VIS/NIR hyperspectral camera, and the changes in maximal quantum yield of PSII photochemistry for a dark-adapted state (Φ_Po_, also referred as F_V_/F_M_) reflecting the photosynthetic activity of the plants obtained from KCFIM.

Apart from the importance of root-growth analysis, a key descriptive parameter in plant physiology is the growth of plant shoots. Although there are numerous secondary traits describing the morphology of shoots in particular species and their developmental stages, the primary and universal trait is biomass formation. Shoot biomass is defined as the total mass of all the aboveground plant parts at a given point in a plant’s life [8]. This trait can be easily assessed by a simple weighing of the fresh (FW) and dry (DW) masses. However, this involves the destruction of the measured plant thus only allowing end-point analyses. Similarly, leaf area and consequently the plant growth rate are usually determined by manual measurements of the dimensions of plant leaves [9-11]. Such measurements are highly time consuming and thus cannot be used for large scale experiments. For this reason, plant phenotyping facilities prefer to evaluate the growth rate using imaging methods which employ digital cameras with subsequent software image analysis. This enables a faster and more precise determination of the leaf area [12-14] and other parameters called projected area (Figure 2), or hull area in the case of monocots [15,16]. In general, non-invasive techniques of shoot growth determination have proven very reliable, and high correlations between the digital area and the shoot fresh, or dry weights, respectively, were reported in *Arabidopsis*, tobacco [17], cereals [18,19], and pea [20]. An example of a general shoot phenotyping protocol based on biomass estimation was reported by Berger et al. [21]. Similarly, other common morphometric parameters such as stem length, number of tillers and inflorescence architecture can be assessed non-destructively and manually, but again the time requirements, limit the number of plants analysed. High-throughput approaches for analyses of these rather species-specific traits would be very valuable [15], however, with the exception of *Arabidopsis* [22] the range of accessible solutions is still limited (for some emerging methods see [23-26]).

Correct determination of digital plant growth area can be distorted by overlapping leaves, leaf twisting and curling, and circadian movement, especially when the RGB image is taken only from one view (e.g. from top view). A new approach developed for *Arabidopsis* consisting of plant area estimation (which takes into account leaf overlapping), growth modelling and analysis, followed by application of a nonlinear growth model to generate growth curves, and subsequent functional data analysis, was shown to analyse the plant growth in high-throughput experiments more precisely [14]. However, due to the use of only a top-view RGB imaging, this approach cannot be applied for analyses of most of the agronomical important plants with vertical growth. A set-up that introduces more projections (e.g. side-views) into the phenotyping platforms thus can partially solve this problem. The three-views RGB imaging together with linear mathematical modelling was used for accurate estimation of plant shoot dry weight of wheat and barley from two dimensional images [18]. The accuracy of three-view approach has been recently validated in species with challenging shoot morphology such as field pea [20].

#### Chlorophyll fluorescence imaging (CFIM)

One of the chlorophyll (Chl) fluorescence methods is chlorophyll fluorescence induction (CFIN), i.e., the measurement of the Chl fluorescence signal during illumination of the sample following prior dark adaptation. Since the first paper on CFIN by Kautsky and Hirsch [27], CFIN has been one of the most common methods used in photosynthesis and plant physiology research: it is inexpensive, non-destructive, and above all, provides a great deal of information about the photosynthetic function of the sample (reviewed, e.g., by Lazár [28,29]). Use of pulse amplitude modulation (PAM) techniques for the measurement of CFIN together with the application of the saturation pulse (SP) method enables the separation of photochemical and non-photochemical events occurring in the sample [30]. Chl fluorescence is excited and measured with the help of weak measuring flashes, whereas photosynthesis is maintained by actinic illumination and saturation of photosynthesis is achieved by the SPs. Since Chls absorb in blue (Chl *a* at 436 nm and Chl *b* at 470 nm, respectively) and red (at about 650 nm for both Chls *a* and *b*) regions of visible spectrum, the measuring and actinic light is the light with one of the above wavelengths, usually 650-nm. The SPs are usually generated by white light. On the other hand, Chl fluorescence emission spectrum at room temperature shows two peaks centred at about 680 and 735 nm. To avoid a possible overlap of the 650-nm excitation light with Chl fluorescence emission, the Chl fluorescence signal is detected at wavelengths longer than 700 nm. To reveal spatial heterogeneity of the fluorescence signal during CFIN, imaging Chl fluorometers were developed [31,32]. In the images (for illustration see Figure 2), different colours are used to show different fluorescence intensities according to a chosen false colour scale (as mentioned above, fluorescence emission is always above 700 nm, red light). An additional advantage of the CFIM is that it provides a huge amount of data which can be thoroughly analysed and used for early detection of plant stress as shown, e.g., by Lazár et al. [33]. At present, modern CFIM instruments adopt PAM and SP methods/techniques and are thus highly suitable for high-throughput plant phenotyping (reviewed, e.g., by Gorbe and Calatayud [34], Harbinson et al. [35]). However, over the course of time, too many Chl fluorescence parameters were defined and claimed to reflect particular functions of photosynthetic apparatus. Hence, there is a problem over which parameter should be measured/evaluated and presented. Values of most of the parameters cannot be mutually compared. It is only possible to compare relative changes (caused, e.g., by a stress treatment) of a given parameter. The parameters of the so-called energy partitioning, i.e., quantum yields of processes responsible for the use of the absorbed light energy, are the best choice (reviewed by Lazár [36]) as they are all defined on the same basis and can be directly compared. Since all quantum yields sum to unity, the quantum yields express fractions of absorbed excitation light that are used for given processes (photochemical and various types of non-photochemical energy dissipations).

It is also worth mentioning here that kinetic types of CFIM (KCFIM) that measure whole CFIN and also apply the SPs which then allow computation of various Chl fluorescence parameters, and integrate signal from the whole leaf or shoot, are the most valuable for physiological studies. However, integration of KCFIM into high-throughput systems [20,37] is not very common and in the majority of recent reports, imaging systems measuring either single Chl fluorescence level (SLCFIM) or two Chl fluorescence levels (usually the minimal and maximal Chl fluorescence levels for the dark-adapted state; TLCFIM) were used (see Table 1). As intensity of Chl fluorescence depends on the amount of chlorophylls, the SLCFIM might be used, e.g. to distinguish between non-stressed and senescent leaves (when the amount of Chls is decreased) at the later stages of stress progression but it does not provide any information about early processes in photosytem II (PSII) that are not necessarily linked to the later senescence events. Further, the usual output of the TLCFIM, the F_V_/F_M_ ratio, which estimates the maximum quantum yield of photosystem II photochemistry, provides only a limited information about photosynthetic function compared with the outputs of the KCFIMs, which also allow determination of the other quantum yields and parameters (see [36] for a review).

#### Thermoimaging

Plants are cooled by transpiration and when the stomata are closed, plant temperature increases. Based on this principle, thermal imaging was used for the first time to detect the changes in the temperature of sunflower leaves caused by water deficiency [38]. In addition to transpiration, stomata also drive water vapour, both parameters being typically determined by leaf gas exchange measurements. However, leaf gasometry involves contact with leaves which often interferes with their function. Further, leaf gasometry is time-consuming, limited by sample size and/or large number of samples required. In addition to heat emission, plants can lose heat by conduction and convection, which in fact represent mechanisms of a non-photochemical quenching of excited states. For this reason, it is not unexpected that an increased thermal signal correlates with an increase in non-photochemical quenching as shown by Kaňa and Vass [39]. Given the foregoing, thermoimaging is a very suitable method for plant phenotyping [19,40,41]. Like CFIM, it uses cameras to measure spatial heterogeneity of heat emissions, usually from leaves; the heat is electromagnetic radiation in the infrared region, usually between 8 – 13 μm. Generally, thermal imaging has been successfully used in a wide range of conditions and with diverse plant species. The technique can be applied to different scales, e.g., from single seedlings/leaves through whole trees or field crops to regions. However, researchers have to keep in mind that environmental variability, e.g., in light intensity, temperature, relative humidity, wind speed, etc. affects the accuracy of thermal imaging measurements and therefore the measurements and their interpretations must be done with care. Although thermal imaging sensors have been integrated into the in-house phenotyping platforms with controlled-environment (see section *The use of phenotyping methods to study plant stress responses*) the majority of studies have been performed so far in field conditions [42-44]. All aspects of thermal imaging used for the exploration of plant-environment interactions, as well as an overview of the application of thermoimaging in field phenotyping, were recently reviewed by Costa et al. [45].

#### Hyperspectral imaging (VIS-NIR, SWIR)

The absorption of light by endogenous plant compounds is used for calculations of many indices which reflect the composition and function of a plant. Such indices are, for example, the normalized difference vegetation index (NDVI) [46], an estimator of the Chl content, and the photochemical reflectance index (PRI) [47], an estimator of the photosynthetic efficiency. The absorption of a compound (e.g., water) at a given wavelength [48] can also be used for direct estimation of the compound contents in the plant. For practical reasons, measurement of absorbance is replaced here by measurements of reflectance. Depending on the measured wavelengths of reflected signal, various detectors are used, usually VIS-NIR (visible-near infrared region (400–750) - (750–1400 nm)) and SWIR (short wavelength infrared region; 1400–3000 nm). Measurements of the reflectance signal in VIS-NIR and SWIR regions originate from methods of remote sensing [49-51]. However, due to the high value of the information they carry, they are very suitable methods for plant phenotyping [52-54]. The reflectance signal can be detected at selected wavelengths or separated spectral bands (so-called multispectral detection). The whole spectral region can also be measured even for each pixel when cameras are applied and the hyperspectral imaging is carried out (Figure 2). Whereas the hyperspectral imaging in the VIS-NIR spectral region is used for evaluation of several indices as mentioned above, the SWIR spectral region is mainly used for the estimation of the plant’s water content. Several aspects of plant reflectance were recently reviewed by Ollinger [55]. Despite the many indices that have been defined so far, based on the reflectance measurements, it is difficult to assess them accurately, similar to the situation with CFIN parameters (see above). For this reason, critical revision of all of the reflectance indices is needed to evaluate which of them provide the required information in the best way.

#### The use of phenotyping methods to study plant stress responses

One of the most important applications of automated plant phenotyping methods is in studies of plants’ responses to various types of environmental stresses. In Table 1 we listed recent reports describing phenotyping protocols developed for indoor automated shoot phenotyping used in stress-related studies. Since the integrative approaches are a logical but rather new step in the development of phenotyping platforms, there are limited reports on the use of simultaneous analysis by multiple sensors. For this reason, we included here “single-sensor” experiments as well, that were performed in the automated platforms.

Perhaps the most widely used application of high-throughput phenotyping is in the search for drought-tolerant varieties. Objectives, traits and approaches related to automated plant selection for drought stress resistance were recently reviewed in Mir et al. [56], and Berger et al. [57]. Here, we add information from examples of the use of non-invasive plant phenotyping in this field. One of the early reports on the use of the high-throughput phenotyping platform describes the employment of the commercial-prototype system for evaluation of drought tolerance in nine *Arabidopsis* accessions [58]. The screening was based on RGB imaging, estimating rosette-leaf area and automated pot weighing and watering to assess transpiration rates. A very similar approach was later used by Skirycz et al. also in *Arabidopsis* [59]. The same platform was further used in a recent physiological study of Clauw and co-authors in which the impact of mild-drought on various *Arabidopsis thaliana* accessions was evaluated [60]. Another study on *Arabidopsis* employing top-view RGB imaging, pot weighing and automated rotation of pots was performed by Tisné et al. [61]. The phenotyping platform was designed to prevent position effect on water evaporation and authors demonstrated important improvement in the evaporation homogeneity [61].

Although these studies represent an important contribution to the development of automated phenotyping, the design of the platform for top-view experiments has limited their use to analyses of plants with leaf rosette. Further progress thus lay in development of platforms allowing RGB imaging from multiple positions. The most recent advances in the use of multiple-view RGB imaging followed by software analysis were demonstrated in a study by Neumann et al. [26]. The authors were able to automatically extract from the images of the barley plants, the plant height and width, and also leaf colours to evaluate the impact of drought on the degradation of chlorophyll. Earlier, Pereyra-Irujo et al. [62], reported a study that employed a self-constructed high-throughput platform for the RGB screening of growth and water-use efficiency (WUE) in two soybean (*Glycine max* L.) genotypes. The system with automated weighing and watering placed in the greenhouse was used to analyse the projected area of the shoots and the mass of the pots [62]. An impressive number of plants was analysed for similar traits in the study by Honsdorf et al. [16]. These authors searched for drought-tolerance QTLs in 48 wild barley introgression lines, using a commercial greenhouse based platform with multiple-view RGB imaging and automated weighing and watering [16]. A similar approach utilizing estimation of shoot biomass based on RGB imaging was used by Coupel-Ledru et al., to screen thousands of grapevine plants for drought tolerance [63]. In these studies, the plant water management was automatically analysed by simple weighing of the pots. This approach, however, begs several questions about the homogeneity of evaporation from the soil of the pots placed in different positions of the growing area. The solution to this issue usually requires an exhaustive validation process with numerous control pots and artificial plant-like objects randomly distributed throughout the growing area (Mark Tester, personal communication). A more elegant solution could be the use of the specific sensors controlling directly the plant water content [64] or transpiration [65] of each plant. Even this approach, however, requires appropriate validation.

An integrative way of analysis was employed in the study of Petrozza et al. [66]. Here, the effect of Megafol treatment on drought-stressed tomatoes was assessed using RGB imaging to distinguish shoot area, SLCFIM measurement to calculate “stress index” and NIR camera for water content estimation. Repeated measurements by NIR camera throughout the experiment allowed visualizing the drop of the high water content index that precedes the growth limitation caused by drought stress [66]. A combination of RGB and NIR imaging techniques was also used by Harshavardhan et al. for analysis of the drought-tolerance of transgenic *Arabidopsis* plants [67]. The RGB imaging was employed by Bresson et al. to study the effect of plant-bacteria interactions on plant tolerance to drought stress [68]. The integration of F_V_/F_M_ measurement by TLCFIM provided complementary information to the growth rate and WUE analysis obtained by pot weighing [69]. A combination of RGB, SLCFIM and NIR imaging techniques was used by Chen et al. [64] to study different phenotypic traits of 18 barley genotypes. The authors used sophisticated statistics and mathematical modelling to classify genotypes based on their response to drought stress [64].

Another important trait in drought studies is the leaf surface temperature that reflects the transpiration rate of the plant (as discussed above in the section *Thermoimaging*). A combination of shoot digital imaging, thermoimaging and automated weighing and watering to study WUE was used by Fehér-Juhász et al. [19]. These authors employed a self-constructed greenhouse-based platform for the selection of drought-tolerant transgenic wheat plants. The platform allows monitoring of the mature cereal plants´ growth by multiple-view RGB imaging and assessment of the leaf surface temperature by side-view thermal camera recording the differences in temperatures of plant shoots [19]. The same platform and a similar phenotyping experimental design were used for evaluation of drought tolerance in barley. The system provides integrative analysis of plant growth and physiology, but its use for large-scale analysis is limited by a semi-automated regime requiring manual loading of the plants into the system [65].

Given that physiological responses to drought and high temperature stresses are tightly connected, similar approaches can be used to study the tolerance of plants to both drought and high temperature. The use of high-throughput phenotyping for high temperature tolerance and a description of the appropriate sensors can be found in a review by Gupta et al. [70]. More recently, the effects of the high temperature on the *Arabidopsis* plants were studied by Vasseur et al. [71]. The authors used commercial-prototype platform allowing the top-view RGB imaging and WUE analysis followed by highly-sophisticated statistical approach to reveal contrasting adaptive strategies to the high temperature and drought stresses [71].

The salinization of soil is another phenomenon often associated with drought and high temperature stress. The example of the protocol for salt stress study in various cereals combining RGB imaging with destructive leaf sampling to measure Na^+^ concentration was described by Berger et al. [72]. The effect of salt stress was studied by Rajendran et al. [73] using digital RGB imaging in a greenhouse-based commercial system. This study provided deep insight into the physiological processes connected with salinity in wheat. The authors used the multiple-view RGB imaging to estimate a digital area of shoot, and to visualize changes in leaf colour for quantification of the senescent area. Using non-invasive plant phenotyping and analysis of Na^+^ concentration in 4th leaf, the authors predicted a plant salinity tolerance index that showed a good correlation with the results obtained from conventional salt-tolerance measurements [73]. Simple RGB imaging in wheat and barley was carried out in the physiological study of Harris et al. [74], and described in the methodological report of Golzarian et al. [18]. Recently, Schilling et al. applied a similar approach to select a salt-tolerant line of transgenic barley [75]. The combination of digital RGB imaging (used to measure shoot growth rate) with SLCFIM (used for the assessment of senescent areas) was used for the selection of salt-tolerant cultivars of rice by Hairmansis et al. [76]. These studies of salt-stress tolerance were performed using the same commercial platform involving SLCFIM sensor. As mentioned in the section *Chlorophyll fluorescence imaging (CFIM)* this type of CFIM in fact provides only estimation of a senescent area that can be obtained using an older way of estimation based on colour detection by RGB imaging. Thus, to increase the value of the physiological evaluation, the use of KCFIM is necessary for quantification of the quantum yield of photochemistry and of the other competitive processes [36].

Combination of RGB imaging, thermoimaging and TLCFIM was used in the pioneer work of Chaerle at al. who evaluated the effects of mild mottle virus infection on tobacco and bean plants [77]. The use of high-throughput techniques in the nutrient starving stress studies have been already reported too. The principle of the method based on RGB imaging of leaf expansion was described by Moreau et al. [78]. A comprehensive study on the phenotypic effects of nitrogen and phosphorus nutrient statuses of *Brachypodium* was carried out by Poire et al. employing RGB imaging to estimate growth rate [79]. A similar approach was used in a study of Neilson et al. [80] where the responses to nitrogen deficiency and drought were evaluated by RGB imaging, NIR imaging and automated weighing, respectively. The authors also developed software that extracted from the images, additive traits such as projected plant height and the height to the ligule of the youngest fully expanded leaf, which showed very good correlations with standard manually measured agronomical parameters [80]. The multiple-sensor approach was described earlier in beans by Chaerle et al., who used RGB imaging, thermoimaging and TLCFIM to evaluate the phenotypes related to magnesium deficiency and biotic stress [81]. The impact of cold stress on plant growth and physiology is routinely studied using non-invasive methods through the analysis of Chl fluorescence, but not using fluorescence sensors integrated into complex growth-analysing platforms [82-84]. Jansen et al. studied the effects of chilling stress in *Arabidopsis* and tobacco plants using a growth chamber based system equipped with digital top-view RGB screening and KCFIM [37]. Very recently an automated screening approach based on RGB imaging and KCFIM analysis for selection of pea cultivars with different cold-sensitivity was developed by Humplík et al. [20]. The reported study was not intended only for selection of cold-sensitive/tolerant varieties of pea but also for studies of plant cold-response strategies in general. Since the CFIM analysis is not limited to plant morphology and the image analysis was sensitive enough to detect tiny tendrils of pea, the described procedure should be theoretically employed for shoot analyses of other plant species [20].

## Conclusions

This mini-review focuses on recent advances towards development of integrative automated platforms for high-throughput plant phenotyping that employ multiple sensors for simultaneous analysis of plant shoots. In both basic and applied science, the recently emerging approaches have found importance as tools in unravelling complex questions of plant growth, development, responses to environment, as well as selection of appropriate genotypes in molecular breeding strategies. As far as phenotype is an interactive network of responses by the plant to its environment that affects in turn, the expression of the genotype it is worth pointing out that attention to the way the analyses are done, under precisely controlled conditions allowing for direct linking the huge amount of complex phenotyping data obtained to the particular conditions. It would also help the end user – the biologist – to narrow his/her view on the importance of various parameters and indices available from the specialized measurements (specifically CFIN and reflectance measurements) and evaluate which of them provide the required information in the best way and hence thus the most suitable for high-throughput plant phenotyping. Such information and standardized protocols applicable for the particular phenotyping methodologies should be available in the near future due to the phenotyping community efforts.

## Abbreviations

Chl: Chlorophyll
CFIM: Chlorophyll fluorescence imaging
CFIN: Chlorophyll fluorescence induction
DW: Dry weight
F_M_: Maximal chlorophyll fluorescence levels for dark- adapted state
FW: Fresh weight
F_V_: variable chlorophyll fluorescence level for a dark-adapted state
Φ_Po_: The maximal quantum yield of photosystem II photochemistry for a dark-adapted state
KCFIM: Kinetic chlorophyll fluorescence imaging
NDVI: Normalized difference vegetation index
PAM: Pulse amplitude modulation
PRI: Photochemical reflectance index
PSII: Photosystem II
RGB: Red-green-blue
SLCFIM: Single-level chlorophyll fluorescence imaging
SP: Saturation pulse
SWIR: Short wavelength infrared
TLCFIM: Two-level chlorophyll fluorescence imaging
VIS-NIR: Visible-near infrared
WUE: Water-use efficiency
